# The Prognostic Role of LRIG Proteins in Endometrial Cancer

**DOI:** 10.3390/cancers13061361

**Published:** 2021-03-17

**Authors:** Zoia Razumova, Husam Oda, Igor Govorov, Eva Lundin, Ellinor Östensson, David Lindquist, Miriam Mints

**Affiliations:** 1 Division of Neonatology, Obstetrics and Gynecology, Department of Women’s and Children’s Health, Karolinska Institutet, 171 77 Stockholm, Sweden; igor.govorov@ki.se (I.G.); ellinor.ostensson@ki.se (E.Ö.); miriam.mints@ki.se (M.M.); 2 Unit of Pathology, Department of Medical Biosciences, Umeå University, 901 87 Umeå, Sweden; husam.oda@umu.se (H.O.); eva.lundin@umu.se (E.L.); 3 Institute of Perinatology and Paediatrics, Almazov National Medical Research Centre, 197 341 St. Petersburg, Russia; 4 Unit of Professional Development, Department of Clinical Sciences, Umeå University, 901 87 Umeå, Sweden; david.lindquist@umu.se; 5 School of Medical Sciences, Faculty of Medicine and Health, Örebro University, 701 82 Örebro, Sweden

**Keywords:** LRIG1, LRIG2, LRIG3, endometrial cancer, prognosis

## Abstract

**Simple Summary:**

Over the past few years, there have been many studies investigating the LRIG (leucine-rich repeats and immunoglobulin-like domains) family of transmembrane proteins, focusing mainly on their role in cancer progression. However, the prognostic role of the proteins in endometrial cancer was not studied, even though it is the most common gynecologic cancer in developed countries. Therefore, our research group aimed to fill this knowledge gap. Here, our study analyzed endometrial cancer tissue using immunohistochemistry. As a result, we found that one of the LRIG proteins—LRIG3—might influence endometrial cancer survival rates. However, the role of the LRIG1 and LRIG2 proteins in the group remains to be clarified. In conclusion, our study expands upon knowledge on the prognostic role of LRIG family in gynecological malignancies.

**Abstract:**

Endometrial cancer (EC) is the most common gynecologic malignancy in Sweden and it has various prognostic factors. The LRIG family is a group of three integral surface proteins with a similar domain organization. The study aimed to explore LRIG family as prognostic factor proteins in EC. The initial study cohort included 100 women with EC who were treated at the Department of Women’s and Children’s Health, Karolinska University Hospital Solna, between 2007 and 2012. We assessed the associations between LRIG protein expression and type, grade, and stage of EC, as well as progression-free and overall survival. Immunohistochemistry results revealed that most women in the analytical sample had >50% LRIG1-, LRIG2- and LRIG3-positive cells. A statistically significant association was observed between having a high number of LRIG3-positive cells and superior overall survival (incidence rate ratio = 0.977; 95% confidence interval: 0.958–0.996, *p* = 0.019). Moreover, positive LRIG3 staining of the cell membrane was associated with reducing in the risk of death (hazard ratio = 0.23; 95% confidence interval: 0.09–0.57). Our results show that LRIG3 expression might be a prognostic factor in EC. The role of LRIG1 and LRIG2 expression remains to be further investigated.

## 1. Introduction

Endometrial cancer (EC) is the most common gynecologic malignancy in developed countries [1]. It occurs mostly in elderly women, with a median age of 71 years at diagnosis. Although the 10-year relative survival rate for women with EC is over 80%, survival rates still vary significantly [2]. There are two histologic categories of EC, type I and type II, which differ in their incidence, clinical characteristics, etc. [3]. Type I EC includes tumors of endometrioid histology and represents about 80% of EC. Type I EC usually has a favorable prognosis, is induced by estrogen, responsive to progestins, and may be preceded by intraepithelial neoplasm [4]. Type II EC accounts for 10–20% of all EC and includes tumors of nonendometrioid histology: serous, clear cell, mucinous, squamous, transitional cell, mesonephric, and undifferentiated.

Endometrioid EC is graded using the International Federation of Gynecology and Obstetrics (FIGO) classification system, which assesses architectural pattern and nuclear grade and is divided into grade 1 (<5% solid growth patterns), grade 2 (6–50% solid growth patterns), and grade 3 (>50% solid growth) [5].

The standard surgery in EC management is total hysterectomy and bilateral salpingophorectomy without vaginal cuff, as claimed by European Consensus on EC. Lymphadenectomy is recommended in patients with high-risk EC [6]. According to Swedish guidelines from 2017, women with EC are divided into high- and low-risk groups. The high-risk group includes women with type II EC, FIGO grade 3, and deep myometrial invasion/cervical stroma invasion according to ultrasound and/or magnetic resonance imaging [7]. All surgical treatment is performed based on the results of a diagnostic endometrial biopsy and radiological examinations, but the subjective nature of histology means that biopsy diagnoses often have poor reproducibility [8]. Moreover, histological features do not show the molecular properties of tumors, like abnormal genetic factors, epigenetic alterations, or environmental factors. However, the heterogenic nature of many primary tumors makes proper molecular diagnosis difficult [8], which may explain why 20% of EC that develop into aggressive and metastatic cancer are first classified as non-aggressive [9]. The molecular characteristics of tumors need to be further investigated in order to address the limitations of the histopathological diagnosis of EC. Moreover, knowledge of the molecular mechanisms of cancer genesis is needed in order to identify potential biomarkers that can be used to treat EC [10].

The leucine-rich repeats and immunoglobulin-like domain (LRIG) family is a group of three integral surface proteins with a similar domain organization. The LRIG peptide contains leucin-rich repeats and immunoglobulin-like domains, a transmembrane glycoprotein, and a cytoplasmic tail [11]. LRIG1 was discovered in 2001 and named Lig-1 (3p14). The locus is often absent in human tumors, including cervical cancer. Interestingly, LRIG1 expression has been found in most human tissues. LRIG2, which is located on 1p13, has the same domain organization as LRIG1 and is also expressed in most human tissues [12]. LRIG3 (12q13) was also found soon thereafter [13]. Analyses of LRIG3 in glioblastoma cells showed that the gene is a potent tumor suppressor, which plays a role in invasion, proliferation, and apoptosis [14].

LRIG1 is underexpressed in malignancies of the skin, cervix, bladder, and lung, but it is overexpressed in astrocytoma, prostate cancer, and lung carcinoid tumors [15]. As LRIG genes could be over- or underexpressed in human tumors, it is important to further investigate the tumor-suppressive or tumor-promoter role of LRIG proteins. As their role in EC is currently unexplored, the present study aimed to assess the prognostic role of LRIG proteins in EC.

## 2. Results

Median age at diagnosis was 68.0 years (IQR 25–75% = 14.0) and median body mass index was 27.1 kg/m^2^ (IQR 25–75% = 8.8), while median parity was 2.0 (IQR 25–75% = 1.3). Approximately half of the women in our analytical sample (53.4%) had used hormone replacement therapy at some point in their life, while only 10.8% had diabetes (Table 1).

Following surgery, 42.7% of women (32/75) underwent radiation and 29.3% (22/75) received chemotherapy. Of the 51 women with type 1 EC, 14 (27.5%) underwent chemotherapy or radiotherapy due to moderate-to-poor differentiation and advanced invasion. Almost all women with type 2 EC (23/24, 95.8%) received adjuvant chemotherapy or radiotherapy. Median age at diagnosis was higher in women who died of EC (72.0, IQR 25–75% = 8.0 vs. 65.5, IQR 25–75% = 17.4; *p* = 0.003), while there was no difference in median BMI or parity.

**Figure 1:**
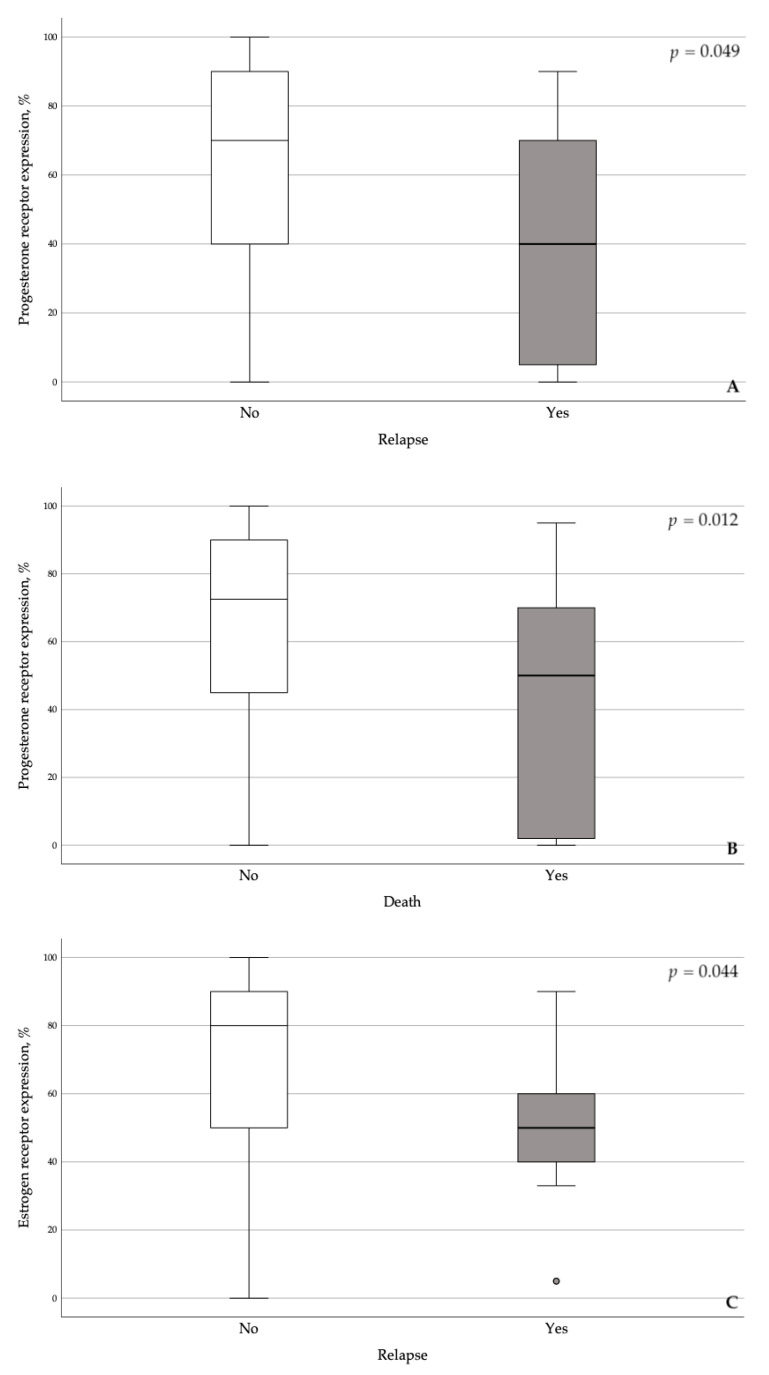

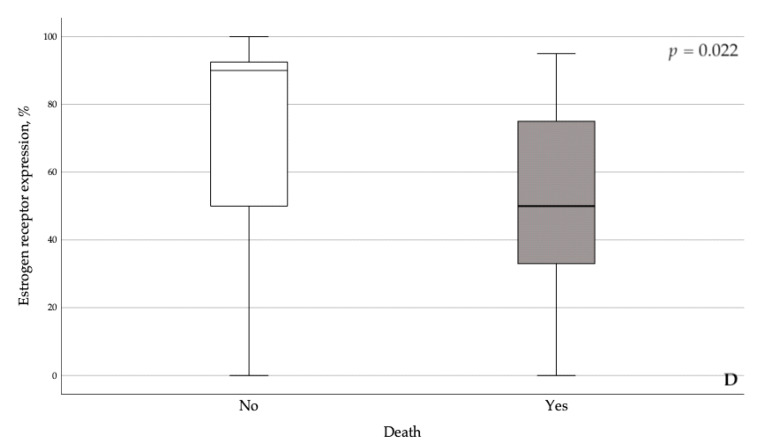
Correlation between progesterone and estrogen receptors expression, and relapse and death.

As expected, the relapse rate was significantly correlated with grade (ρ = 0.306, *p* = 0.008), stage (ρ = 0.538, *p* < 0.0001), and depth of invasion (ρ = 0.301, *p* = 0.009). Incidence of death was also correlated with grade and stage, but not with depth of invasion because the significance level was not reached (ρ = 0.080, *p* = 0.497). Progesterone and estrogen receptors expression were lower in the tumors of women who relapsed or died (Figure 1).

**Figure 2:**
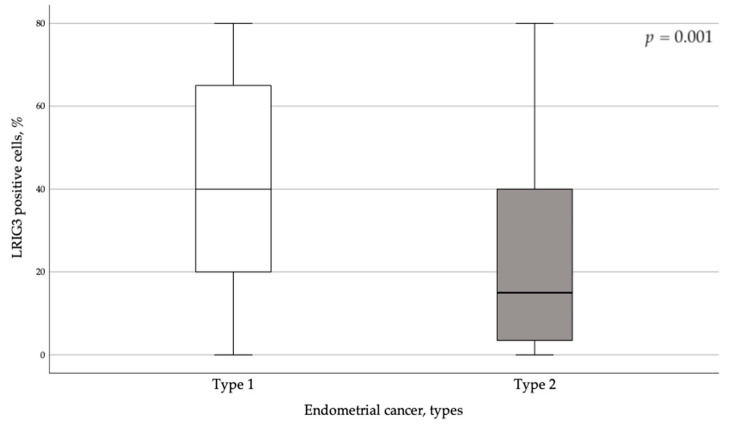
LRIG3 positive cells (%) in different endometrial cancer tumor types.

The majority of women in the cohort had a protein score of 3 for LRIG1 (97.3%) (Table 2). At the same time, LRIG3 was notably underexpressed in type 2 EC (Figure 2).

**Figure 3:**
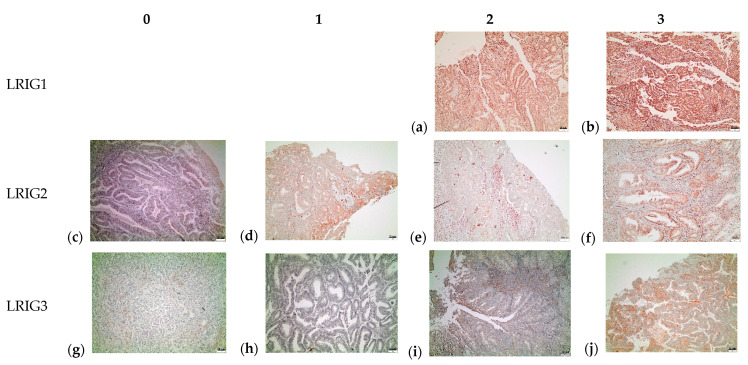
Examples of different protein scores (0 = 0%, 1 = 1–25%, 2 = 26–50%, 3 >50% of positive cells) for LRIG1, LRIG2, and LRIG3 in endometrial cancer, grade 1 (**b**,**c**,**f**,**j**), grade 2 (**d**,**h**,**i**), and grade 3 (**a**,**e**,**g**). Scale bar 20 um.

The different pictures illustrate examples of different protein scores for LRIG1, LRIG2, and LRIG3 in endometrial cancer (Figure 3). Each slide is an example of a representative area. Still, the percentage estimation of positive cells and the FIGO grade is based on evaluation of all tumor cells in that sample.

**Figure 4:**
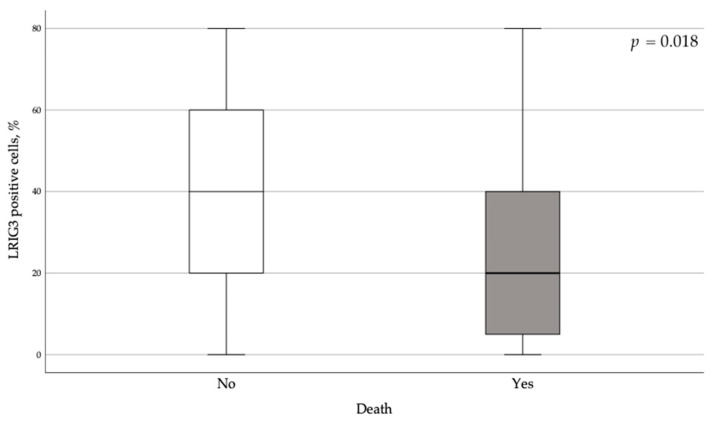
Percentage of LRIG3-positive cells in women who died of endometrial cancer and endometrial cancer survivors.

Among 7570 person-months of follow-up, 14 women relapsed and 23 died. The percentage of LRIG1- and LRIG2-positive cells were not positively or negatively associated with progression-free survival and overall survival. In contrast, LRIG3 expression was two times lower in women who died of EC than in EC survivors (Figure 4).

**Figure 5:**
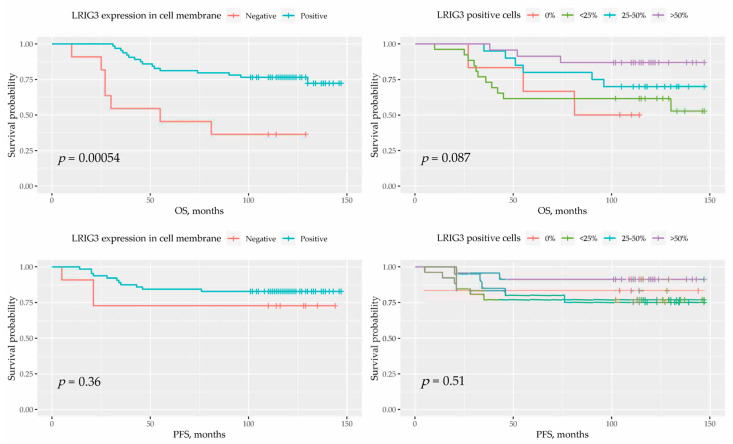
Percentage of LRIG3-positive cells and LRIG3-positive staining of the cell membrane in women who died and in endometrial cancer survivors.

A statistically significant association between a high number of LRIG3-positive cells and superior overall survival was observed (incidence rate ratio = 0.977; 95% confidence interval: 0.958–0.996, *p* = 0.019) (Figure 5).

LRIG3 expression was inversely correlated with ploidy (r = −0.286, *p* = 0.016) and was significantly lower in higher-grade tumors (*p* = 0.009, Kruskal–Wallis test) (Table 3).

At the same time, there were differences in the LRIG3 staining of the cell membrane. Among patients who died, LRIG3 staining was absent in 30.4% of cell membranes, compared to only 7.7% in EC survivors (χ^2^ = 6.59, *p* = 0.01). On the contrary, LRIG3 staining in the nucleus and cytoplasm was similar in these groups. Positive LRIG3 staining of the cell membrane was associated with a reduction in the risk of death (hazard ratio = 0.23; 95% confidence interval: 0.09–0.57, *p* = 0.01) (Figure 4). This effect persisted even after adjustment for age and body mass index (hazard ratio =0.15; 95% confidence interval: 0.04–0.52, *p* = 0.003).

## 3. Discussion

Endometrial cancer is the most common malignancy in females living in developed countries. New data on molecular characteristics of the EC is emerging, which has recently led to implication of the new classification, based on subcellular properties, rather than on clinical data. The new classification aims to stratify patients into more distinct groups with comparable management approaches and prognosis. It also reflects the current tendency towards personalized approach, potentially leading to a better treatment, as it relies on individual molecular features of the tumor. With this work we complement the knowledge on molecular landscape of the EC by reporting the differences in expression of the proteins embraced within LRIG-family.

In the current study, we aimed to evaluate LRIG protein expression as a prognostic marker in patients with EC. The EC biopsies of almost all women had a protein score of 3 (>50% positive cells) for LRIG1. A high number of LRIG3-positive cells was associated with superior overall survival, and positive LRIG3 staining of the cell membrane was associated with a reduced risk of death. Low LRIG3 expression was also associated with aneuploidy and higher-grade tumors. However, expression of LRIG1 and LRIG2 did not correlate with survival.

Leucine-rich and immunoglobulin-like domains family consists of three proteins (LRIG1, LRIG2, LRIG3) that are widely expressed in different human tissues [13,14,15,16]. They modulate growth factor receptors and signaling. Despite this, the downregulated genes are often deleted in human tumors [17]. At the molecular level, the encoded transmembrane protein LRIG1 acts as a negative regulator of growth factor signaling [18,19]; the LRIG2 protein works as a tumor promotor [20,21,22]; and the LRIG3 protein functions as a tumor suppressor [23,24]. Nowadays, a lot of information is being collected on the role of LRIG proteins in human tumors, and there are published studies on the prognostic role of LRIG proteins in different gynecologic malignancies, such as cervical cancer, vaginal carcinoma, and vulvar squamous cell carcinoma [20,23,25,26,27,28]. However, no data are available on the influence of these proteins on the prognosis of EC. Therefore, to the best of our knowledge, our study is the first to show this prognostic role.

Although LRIG1 and LRIG3 work as tumor suppressors, progression-free survival and overall survival were not associated with LRIG1 expression in our study sample. Although a larger sample size may elucidate additional findings, taking into account predominantly high LRIG1 expression and intensity, we hypothesize that our results may persist even in a larger study sample. Although LRIG2 has been shown to be highly downregulated in endometrial adenocarcinoma cell lines [29], the present study did not identify any prognostic role of LRIG2 expression in EC. This could be the result of a different cellular context or tumor microenvironment in tissue samples in comparison with the mentioned cell lines, but the size of the cohort may also be too small to identify a real difference.

The long-term follow-up of our study sample confirmed the positive prognostic role of LRIG3 expression in EC. Several mechanisms could explain this finding. First, it was previously suggested that LRIG3 overexpression could arrest the cell cycle in the G(0)/G(1) phase, and induce apoptosis in cell lines of glioma, prostate cancer, and bladder cancer [30,31]. It was also shown that LRIG3 could negatively control the ERK pathway, which is known to be often upregulated in human cancer [24,32,33]. LRIG3 was cloned by Guo et al. in 2004 [15]. This protein coding gene, located on chromosome 12q13.2, contains 19 exons. LRIG3 is mainly localized in cytosol and shows low tissue specificity [12].

Interestingly, LRIG3 expression in the cell membrane was associated with a reduced risk of death. This may be valuable information, since it was previously found that LRIG3 is mostly expressed in cytosol, despite its transmembrane glycoprotein origin [11]. The strength of the current study is its long follow-up period. We were able to compare pathological data obtained 10 years ago with current clinical outcomes. The semi-quantitative evaluation of IHC staining may have led to bias, but this could have been overcome by automized assessment.

## 4. Materials and Methods

### 4.1. Study Population and Analytic Cohort

One hundred women who underwent hysterectomy and bilateral salpingoophorectomy as treatment for EC at the Karolinska University Hospital (Solna, Sweden) between 2007 and 2012 were eligible for inclusion in the present analysis. In accordance with current clinical guidelines, some women also received adjuvant treatment at Karolinska University Hospital. EC biopsies were collected during surgery, and a total of 25 women were excluded due to insufficient EC material. Thus, 75 women were included in the final analytical sample.

Routines to include a patient into the study group were as follows. Women who either had symptom suggestive of endometrial cancer or those who met diagnostic criteria were referred to Karolinska University Hospital (Solna, Sweden) for further management. In accordance with current clinical guidelines, the mainstay of the endometrial cancer treatment is surgery. Therefore, women scheduled for hysterectomy and bilateral salpingo-ophorectomy were invited to participate. They were provided with a study description and informed consent. Inclusion covered women undergoing surgery in the time-period 2007–2012. The exclusion criteria were based on the clinical features and some women also underwent adjuvant treatment at Karolinska University Hospital. One hundred women who underwent treatment for endometrial cancer at the Karolinska University Hospital Solna between 2007 and 2012 were eligible for inclusion in the present analysis. In accordance with current clinical guidelines, some women also received adjuvant treatment at Karolinska University Hospital. Endometrial cancer biopsies were collected during surgery and further processed as described below. It is noteworthy that a total of twenty-five women were excluded due to insufficient biological material. As a result, seventy-five women were included in the final analytical sample.

### 4.2. Endometrial Cancer Biopsies and Other Biologic Samples

Following surgery, hospital personnel fixed EC biopsies in a neutral buffered 4% formaldehyde solution, dehydrated, and embedded in paraffin. Serial sections were cut at a thickness of 5 μm and stained with hematoxylin/eosin for further pathological evaluation.

All cases were stained immunohistochemically using the following primary antibodies: estrogen receptor clone SP1, Ventana ready-to-use 790-4325; progesterone receptor clone IE2, Ventana ready-to-use 790-4296. All stains were performed on a Ventana XT platform (Ventana Medical Systems, Tucson, AZ, USA), using locally validated protocols. The stains were evaluated in the following manner: brown nuclei were considered positive regardless of staining intensity; the percentage of positive tumor cells was registered; a cutoff value of 10% was used to define receptor positivity.

Blood samples and adjacent normal tissue biopsies were also taken for each woman and stored at the hospital.

### 4.3. Immunohistochemistry

Five-micrometer thick sections from formalin-fixed, paraffin-embedded biopsies were obtained from archival tissue. After sectioning, they were deparaffinized, rehydrated, and rinsed in water. The Ventana standard procedure was used for immunohistochemical staining in a Ventana BenchMark ULTRA (Ventana Medical Systems, Tucson, AZ, USA). CC1 buffer was used for antigen retrieval. The following antibodies were used: rabbit anti-LRIG1-Vina 16, 22 µg/mL; rabbit anti-LRIG2-151 14, 3 µg/mL; rabbit anti-LRIG3 207-221 34, 8.8 µg/mL.

### 4.4. Evaluation and Classification of Immunostaining

Immunostainings were evaluated blindly by two independent senior pathologists (HO and EL). Both staining intensity and percentage of immunoreactive epithelial cells were assessed within the tumor. A four-level semi-quantitative scale was used to grade intensity as follows: no staining, weak intensity, moderate intensity, or strong intensity. The percentage of immunoreactive cells was also evaluated semi-quantitatively as follows; 0 (0% positive cells), 1 (1–25% positive cells), 2 (26–50% positive cells), and 3 (>50% positive cells).

### 4.5. Medical Records

Take Care is an e-health software used to handle electronic medical records in many Swedish regions, including Stockholm County. The following information was collected from Take Care for each included woman: detailed histopathological analysis of the tumor and detailed medical history (Table 1). The latest update of the data used in this analysis was performed in April 2020.

### 4.6. Ethics Statement

To minimize the integrity violation, we unidentified all patients after data were collected.

### 4.7. Statistical Analyses

Due to predominantly non-normal distribution, we used the median and interquartile range (IQR) to report the central tendency. We also used Pearson chi-square or Fisher’s exact test to find differences within sets of categorical variables and the Mann Whitney U test/Kruskal–Wallis to compare the continuous variables’ distributions. The time-to-event was set at EC relapse, death from any reason or 1st of April 2020, depending on which occurred first. The time period from EC diagnosis to relapse was defined as progression-free survival (PFS), to death of any cause—as overall survival (OS). To illustrate the event’s probability over the time interval, we used the Kaplan–Meier curve and log-rank test to compare survival distribution. Variables that differed significantly in univariate analysis were included in the Cox regression multivariate analysis.

The significance level was set at 0.05. We used IBM SPSS 27.0 (IBM Corp., Armonk, NY, USA) and RStudio 1.3.1093, running on Mac OS.

## 5. Conclusions

The results obtained in this study demonstrate that LRIG3 expression may have a prognostic role in women with EC. Additionally, in the era of individualized medicine, we would like to encourage further assessment of the use of LRIG3 in clinical settings, both during diagnostics and during the management of patients with EC. The significance of LRIG1 and LRIG2 remains to be clarified.

## Figures and Tables

**Table 1:** Characteristics of the analytical sample at endometrial cancer diagnosis and characteristics of corresponding endometrial cancer biopsies (*n* = 75).

Characteristic	Range	Cohort (*n* = 75)	Type 1 (*n* = 51)	Type 2 (*n* = 24)
Age at EC diagnosis (years)	-	66.9 (42–92)	66.3 (42–87)	68.1 (44–92)
Body mass index (kg/m^2^)	-	28.4 (18.4–47.3)	28.3 (20.5–47.3)	28.6 (18.4–36.5)
Parity (%)	None	13.3	13.7	12.5
	1–2	61.3	66.7	50
	≥3	24	19.6	33.3
Diabetes (%)	n/a	10.7	12	9
Hormone replacement therapy—ever use (%)	n/a	52	51	59
Type of EC (%)	Endometrioid adenocarcinoma	37.8	n/a	n/a
	Serous carcinoma	0	n/a	n/a
	Clear cell carcinoma	8.1	n/a	n/a
	Sarcoma or myosarcoma	8.1	n/a	n/a
	Mixed cell type	45.9	n/a	n/a
Grade of EC (%)	G1 (high)	29.3	43.1	0
	G2 (moderate)	32.0	47.1	0
	G3 (poor)	38.7	9.8	100.0
Stage of EC (%)	IA	65.3	88.2	16.7
	IB	18.7	11.8	33.3
	II	4	0	12.5
	IIIA	5.3	0	16.7
	IIIB	1.3	0	4.2
	IIIC (IIIC1 or IIIC2)	1.3	0	4.2
	IVA	0	0	0
	IVB	4	0	12.5
Depth of invasion (%)	None	9.3	11.8	4.2
	<50%	65.3	76.5	41.7
	≥50%	25.3	11.8	54.2
Ploidy (%)	Diploid	62.7	88.2	8.3
	Aneuploid	36	11.8	87.5
p53	%	24	10	57
Progesterone receptor expression	%	57	71.3	27.3
Estrogen receptor expression	%	64.5	77.9	36.5

**Table 2:** Immunohistochemical staining for LRIG1, LRIG2, and LRIG3 (*n* = 75).

Protein	Protein Score *	*N* (%)	Type 1	Type 2
LRIG1	0	0	0	0
	1	0	0	0
	2	2 (2.7)	1 (2)	1 (4.2)
	3	73 (97.3)	50 (98)	23 (95.8)
LRIG2	0	1 (1.3)	1 (2)	0
	1	34 (45.3)	24 (47.1)	10 (41.7)
	2	22 (29.3)	13 (25.5)	9 (37.5)
	3	18 (24.0)	13 (25.5)	5 (20.8)
LRIG3	0	4 (5.3)	1 (2)	3 (12.5)
	1	29 (38.7)	16 (31.4)	13 (54.2)
	2	25 (33.3)	18 (35.3)	7 (29.2)
	3	17 (22.7)	16 (31.4)	1 (4.2)

* Protein score: 0 = 0%, 1 = 1–25%, 2 = 26–50%, 3 > 50% of positive cells.

**Table 3:** Characteristics of the analytical sample at endometrial cancer diagnosis and characteristics of corresponding endometrial cancer biopsies (*n* = 75).

	Type		Grade		
	1	2	1	2	3
LRIG1	90.00 (80.00–95.00)	90.00 (80.00–95.00)	90.00 (85.00–95.00)	90.00 (80.00–95.00)	90.00 (80.00–95.00)
	*p* = 0.755		*p* = 0.795		
LRIG2	30.00 (10.00–55.00)	30.00 (15.00–50.00)	15.00 (5.00–50.00)	30.00 (15.00–45.00)	35.00 (10.00–65.00)
	*p* = 0.657		*p* = 0.293		
LRIG3	40.00 (20.00–65.00)	15.00 (2.75–40.00)	45.00 (20.00–65.00)	35.00 (17.50–55.00)	10.00 (1.00–40.00)
	*p* = 0.001		*p* = 0.001		

## Data Availability

The datasets used and analyzed during the current study are available from the corresponding author on reasonable request.
